# In situ NMR reveals real-time nanocrystal growth evolution via monomer-attachment or particle-coalescence

**DOI:** 10.1038/s41467-020-20512-6

**Published:** 2021-01-11

**Authors:** Reut Mashiach, Haim Weissman, Liat Avram, Lothar Houben, Olga Brontvein, Anna Lavie, Vaishali Arunachalam, Michal Leskes, Boris Rybtchinski, Amnon Bar-Shir

**Affiliations:** 1 Department of Organic Chemistry, Weizmann Institute of Science, Rehovot, 7610001 Israel; 2 Department of Chemical Research Support, Weizmann Institute of Science, Rehovot, 7610001 Israel; 3 Department of Materials and Interfaces, Weizmann Institute of Science, Rehovot, 7610001 Israel

**Keywords:** Materials chemistry, Nanoscale materials, Nanoparticles, NMR spectroscopy

## Abstract

Understanding inorganic nanocrystal (NC) growth dynamic pathways under their native fabrication environment remains a central goal of science, as it is crucial for rationalizing novel nanoformulations with desired architectures and functionalities. We here present an in-situ method for quantifying, in real time, NCs’ size evolution at sub-nm resolution, their concentration, and reactants consumption rate for studying NC growth mechanisms. Analyzing sequential high-resolution liquid-state ^19^F-NMR spectra obtained in-situ and validating by ex-situ cryoTEM, we explore the growth evolution of fluoride-based NCs (CaF_2_ and SrF_2_) in water, without disturbing the synthesis conditions. We find that the same nanomaterial (CaF_2_) can grow by either a particle-coalescence or classical-growth mechanism, as regulated by the capping ligand, resulting in different crystallographic properties and functional features of the fabricated NC. The ability to reveal, in real time, mechanistic pathways at which NCs grow open unique opportunities for tunning the properties of functional materials.

## Abstract (web summary)

Understanding nanocrystal growth pathways under their native fabrication environment remains a central goal of science. By synthesizing nanofluorides under in-situ NMR conditions, the authors are able to probe their sub-nm growth evolution, elucidating their formation by coalescence or monomer-attachment.

## Introduction

Colloidal inorganic nanocrystals (NCs) have revolutionized science thanks to their provision of a controllable synthetic platform for tackling fundamental challenges in materials science and their utility in a variety of applications, from catalysis and renewable energy to nanomedicine^1–6^. Their exceptional features (e.g., optical, magnetic, chemical, catalytic, and electrical) often stem from their composition, size, and morphology, emphasizing the essentialness of mechanistic knowledge on NC formation from solution for strategizing novel fabrications. The relationships between NC growth pathways and resultant architectures and functions were revealed thanks to the development of a variety of ex situ and in situ methodologies^7^. Both spectroscopy- and microscopy-based techniques allow us to monitor NCs’ size evolution, alternations in chemical composition, crystallography properties, and change in shape throughout the course of their formation^8–19^. However, they often require temporal reaction sampling, destructive-energy radiation applications, the use of out-of-equilibrium conditions (e.g., drying, freezing, heating, vacuum) and custom-made apparatuses. Thus, there is a need to implement accessible, alternative in situ approaches that can be performed under authentic nanofabrication settings.

Fluoride-based NCs (i.e., nanofluorides) have garnered much attention recently due to their potential use in optics, catalysis, optoelectronics, bioimaging, superconductor devices, batteries, and lubricants^20^. Amongst their advantages over other inorganic NCs, their low phonon energies and high chemical stability stand out, making nanofluorides one of the preferable host-materials for upconversion fluorescence^21–26^. These unique characteristics are governed by their crystallinity, size, shape, and introduced impurities, which are strictly regulated by their formation pathways^27–29^. Interestingly, the rich fluoride content and relatively small-size of colloidal nanofluorides result in a characteristically high-resolution ^19^F-NMR spectrum while in solution^30^. This property has been exploited to design water-dispersed CaF_2_ NCs as nanotracers for in vivo ^19^F-MRI applications, but also provides unprecedented opportunity to use nanofluorides as a platform for tracking NC formation in real time with a high-resolution (HR) NMR setup.

The unique capabilities of NMR-based approaches, which allow the monitoring of changes in the chemical environment of a variety of NMR active nuclei, offer a versatile tool for studying growth pathways and kinetics, surface chemistry, and the physical properties of both organic and inorganic materials^31–36^. In particular, the spectral resolution of NMR shows promise for the characterization of nanomaterials by way of detecting both the organic ligands^37^ and inorganic core elements^38^ as a means to explore their formation in solution. Moreover, the ability to spectrally resolve surface from bulk atoms via NMR^39–43^ makes this method an attractive way to characterize inorganic NC architecture.

Inspired by these studies, we herein present a high-resolution liquid-state NMR-based approach for studying the dynamics of NCs’ size evolution in a quantitative manner without disturbing the reaction conditions (e.g., avoiding sampling, destructive radiation, unwanted heating, undesired freezing, drying, vacuum, etc.). By synthesizing nanofluorides under in situ NMR conditions, and based on the ability to detect the ^19^F-NMR signals of MF_2_ (*M* = Ca^2+^ or Sr^2+^) in water, we are able to probe their sub-nm growth over the entire course of their formation, highlighting their controllable growth mechanisms (coalescence vs. classical simple grow), which result in different morphological and functional features.

## Results

### HR-NMR accurately determines the diameter of NCs in solution

**Fig. 1:**
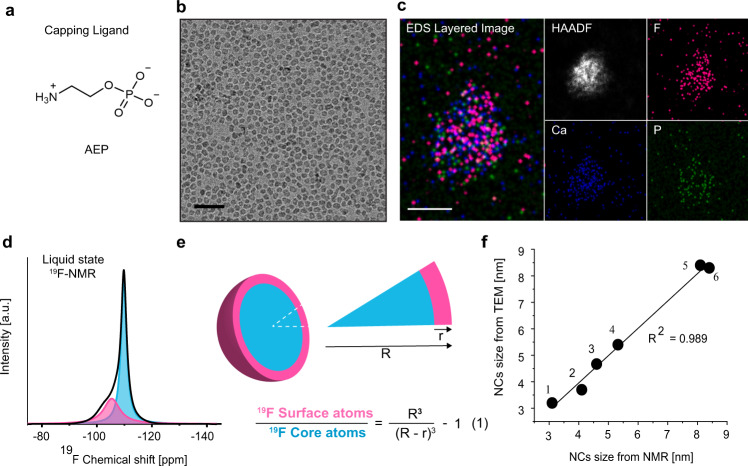
Characterization of AEP-CaF_2_ NCs. **a** Molecular structure of an AEP ligand at pH 7. **b** Cryo-TEM AEP-CaF_2_ NCs (scale bar 20 nm). **c** STEM-EDS elemental maps of an agglomerate of dried AEP-CaF_2_ NCs (scale bar 10 nm). Shown are a combined map of the NCs elements, a high-angle annular dark-field (HAADF) image (white) and elemental maps of each element, i.e., F (pink), Ca (blue), and P (green). (**d**) Liquid-state, high-resolution ^19^F-NMR spectrum of water-dispersed AEP-CaF_2_ NCs and its deconvoluted signals, attributed to ^19^F atoms at the surface of the NCs (-105 ppm, pink) and in the NC core (−109 ppm, light blue). **e** Schematic illustration of the CaF_2_ NC with its surface (pink) and core atoms (light blue) and the equation (1) used to evaluate the size of NCs from the ^19^F-NMR spectrum (Eq. (1) and Supplementary Note 1). **f** Correlation between the sizes measured with NMR and TEM of various CaF_2_ and SrF_2_ NCs bearing different ligands (AEP, Citrate (Cit), Oleic Acid (OA) and Polyethylene Glycol (PEG)); (1) Cit-CaF_2_ (Fig. 5b) (2) AEP-CaF_2_ (3) AEP-SrF_2_ (Fig. 3) (4) OA-SrF_2_ (Supplementary Fig. 3b) (5) PEG-CaF_2_ (Supplementary Fig. 3a) (6) OA-CaF_2_ (Supplementary Fig. 3c).

Prior to conducting HR-NMR studies on water-dispersed, small-sized CaF_2_ NCs capped with 2-aminoethyl phosphate (AEP, at pH7, molecular structure, Fig. 1a), they were synthesized and fully characterized with complementary techniques. Cryo-TEM images revealed monodispersed NCs with an average size of 3.7 ± 0.6 nm (Fig. 1b and Supplementary Fig. 1a) with the confirmed composition of calcium, fluoride, and phosphate (from the ligand), as evaluated by high-angle annular dark-field (HAADF) STEM imaging and energy dispersive X-ray spectroscopy (EDS) (Fig. 1c). The fluorite, faced cubic centered (fcc), crystal structure of the obtained AEP-CaF_2_ NCs was confirmed by powder X-ray diffraction (XRD, Supplementary Fig. 1b) and their relatively small size as well as their monodispersity were preserved in water, as revealed by dynamic light scattering (DLS, Supplementary Fig. 1c).

The high-resolution ^19^F-NMR studies of the synthesized AEP-CaF_2_ NCs in water resulted in a ^19^F-NMR spectrum, from which two populations of fluorides could be detected, at −105 and −109 ppm, assigned to fluorides at the surface and core of the NCs, respectively^44,45^ (Fig. 1d). This assignment was further supported by the significant paramagnetic effect on the ^19^F-NMR peak attributed to the fluorides on the surface of the formulation (Supplementary Fig. 2) emphasizing one of the key features of NMR to provide detailed information that reflects the chemical environment of constituent nuclei. Indeed, NMR studies of nuclei within the NCs (rather than nuclei of the coating ligands) can distinguish between the nuclei at the NC’s solid core and those located at the outer surface, due to differences in chemical environments, relaxation properties, and crystallinity characteristics^39–43^.

We next deconvolved the obtained ^19^F-NMR spectrum for AEP-CaF_2_ NCs, using computational line fitting to quantify the ratio between the ^19^F-atoms at the surface of CaF_2_ NCs (Fig. 1d, pink) and the ^19^F-atoms at the core of CaF_2_ NCs (Fig. 1d, light-blue). From this ratio and by using Eq. (1) (Fig. 1e, Supplementary Note 1), we could calculate the average diameter of the AEP-CaF_2_ NCs in water, which was found to be 4.1 nm (Fig. 1d). This ability to quantify the average diameter of water-dispersed nanofluorides from their HR-^19^F-NMR spectrum by classification and quantification of surface and core atoms provides a unique approach for evaluating the average size of NCs while in solution.

To demonstrate the robustness and reproducibility of this approach, we applied it to evaluate the sizes of various nanofluoride (CaF_2_ or SrF_2_) fabrications (Fig. 1f and Supplementary Fig. 3) from their HR ^19^F-NMR spectra and found them to be in good correlation (*r*^*2*^ = 0.989) with the sizes obtained from HR-TEM images. This ability to accurately determine the average size of a variety of small-sized NCs demonstrates not only the uniqueness but also the potential variability of the proposed HR-NMR-based method. Importantly, magic-angle spinning (MAS) solid-state ^19^F-NMR of the same synthetic AEP-CaF_2_ colloids’ powder showed a similar ^19^F-NMR spectrum (Supplementary Fig. 4a) to that obtained using high-resolution ^19^F-NMR (Supplementary Fig. 4b), from which similar NC diameters were calculated. This observation confirms that water-dispersed AEP-CaF_2_ NCs tumble fast enough in solution so as to average out the intrinsic homonuclear dipolar interactions, allowing to directly detect their fluoride content with HR liquid-state ^19^F-NMR setups.

### Observing NC growth in real time with HR-NMR

The ability to evaluate NC sizes at nanometer scale directly from solution without disturbing the system, using the HR 1D-NMR spectrum, offers a platform to monitor, in real time, NC growth dynamics. We validated this possibility by synthesizing nanofluorides in an NMR tube under ambient conditions while acquiring consecutive ^19^F-NMR spectra from reaction initiation to final formation, monitoring simultaneously the size of the NCs, their concentration at each timepoint of the reaction and the consumption of the F^-^ anions. Controlling the reaction conditions allowed to shed light on the dynamics of NC formation, highlighting their growth mechanism.

To synthesize the NCs, we started by adding a solution of Ca^2+^ cations to an aqueous reaction solution containing both F^-^ anions and AEP capping ligands.

**Fig. 2:**
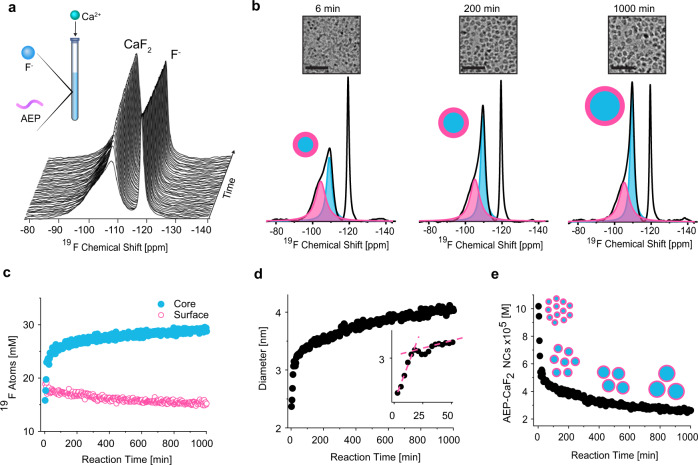
Real-Time, high-resolution ^19^F-NMR of CaF_2_ NC evolution in water. **a** Stack plot of real-time ^19^F NMR spectra after the addition of Ca^+2^ to the aqueous solution of AEP and F^-^ anions. Shown spectra were taken every 30 min (experimental temporal resolution, 3 min). **b** Deconvoluted ^19^F-NMR spectra from three different time points during the reaction (6, 200, and 1000 min), showing the signals of ^19^F atoms on the surface (pink) and in the core (blue). Upper images are the correspondent cryo-TEM images of AEP-CaF_2_ NCs. **c** Quantitative plot of the ^19^F-NMR signals of the fluoride atoms in the core and on the surface of the formed NCs, calculated from the convoluted peaks’ areas at −109 ppm and −105 ppm, respectively. **d** Evolution of the AEP-CaF_2_ NC diameter in water throughout the reaction process. Diameters were derived from Eq. (1). Inset shows the first 50 min of the reaction; dashed lines are a visual guide for the two respective slopes of the NC evolution stages. **e** The concentration of AEP-CaF_2_ NCs (multiply by 10^5^ in the graph)  in the reaction solution as a function of reaction time, calculated from Supplementary Equation 2.

The NMR tube was then placed in a 9.4T NMR spectrometer and sequential ^19^F-NMR spectra were acquired to follow the temporal formation of CaF_2_ colloids, resulting in a ^19^F-NMR stack plot (Fig. 2a). Our results showed that each ^19^F-NMR spectrum consisted of two main distinct peaks that could be, simultaneously, identified and assigned. One peak resonated at −120 ppm, assigned to the free F^-^ anion, indicating its consumption with time (Supplementary Fig. 5). The second downfield ^19^F-NMR peak was attributed to the fluoride content of the CaF_2_ NC, from which the NC’s diameter could be determined using the approach outlined in Fig. 1. Representative line fittings of the experimental ^19^F-NMR spectra obtained at three different time points after reaction initiation (i.e., 6, 200, and 1000 min) are presented in Fig. 2b, depicting the varying ratio between the two ^19^F-spin populations of NCs, which reflects their size evolution. Representative cryo-TEM images of the reaction solution at the same reaction time points (i.e., 6, 200, and 1000 min) are shown above each spectrum in Fig. 2b. The amounts of ^19^F-atoms on the NC surface (pink) and in its core (light blue) were quantified and plotted as a function of the reaction time (Fig. 2c, 3 min temporal resolution). The diameter of the CaF_2_ NCs at each timepoint was then derived from each individual ^19^F-NMR spectrum (using Eq. (1) and Supplementary Note 1) and plotted as a function of reaction time (Fig. 2d). From that plot, a notably rapid formation (3 min after reaction initiation) of ultrasmall CaF_2_ NCs (2.3 nm) is observed, followed by a period of relatively fast growth (up to 3.2 nm within the first 20 min), which then gives way to much slower growth dynamics. In the regime of rapid NCs growth, the ^19^F-NMR resonance of the surface atoms was significantly affected, as expected for smaller NCs that display a larger curvature (Supplementary Fig. 6).

Intriguingly, a mild but pronounced decrease in NC diameter was observed between 20 and 30 min (Fig. 2d inset) that is followed by a continuous growth, a phenomenon that could indicate coalescence events during NC growth^10,46,47^. In parallel to the fast growth rate of the NCs (first 20 min, Fig. 2d), a significant reduction in their concentration was noted (first 20 min, Fig. 2e and Supplementary Equation 2), which was followed by a milder decrease in their total concentration. This observation that numerous small NCs transform into larger (but fewer) NCs, together with the detected intermediate stage (Fig. 2d inset), supports the notion that AEP-CaF_2_ NCs predominantly grow through coalescence^7,48^, a conclusion that is in good agreement with the “coalescence point” that can clearly be seen in the inset of Fig. 2d and found to be reproducible (*N* = 4, Supplementary Fig. 7). Such coalescence events were also captured by both cryo-TEM (Supplementary Fig. 8) and high-resolution TEM (Supplementary Fig. 9) images sampled 25 min from the reaction initiation. Such a mechanistic pathway is promoted by the unfavorable thermodynamic state of initially formed ultrasmall NCs^10^, together with a relatively high number of NCs in solution, which may increase their collision probabilities and, essentially, lead to their coalescence to form larger, more stable NCs. Note here that Ostwald ripening mechanistic pathway may also occur but it is probably much less prominent than coalescence for AEP-CaF_2_ NCs growth, as reflected in both in situ NMR spectroscopy (Fig. 2 and Supplementary Fig. 7) and TEM imaging (Supplementary Fig. 8 and Supplementary Fig. 9). Importantly, we further validated the temporal change in the size of the NCs over time with ex situ cryo-TEM. To that end, the reaction mixture was sampled at three different stages (6 min, 200 min, and 1000 min; Fig. 2b and Supplementary Fig. 10). The NC diameters obtained from cryo-TEM images (Supplementary Fig. 10e) were in a very good correlation (Supplementary Fig. 10f, *R*^*2*^ = 0.98) with those calculated from in situ ^19^F-NMR at the same reaction stages.

**Fig. 3:**
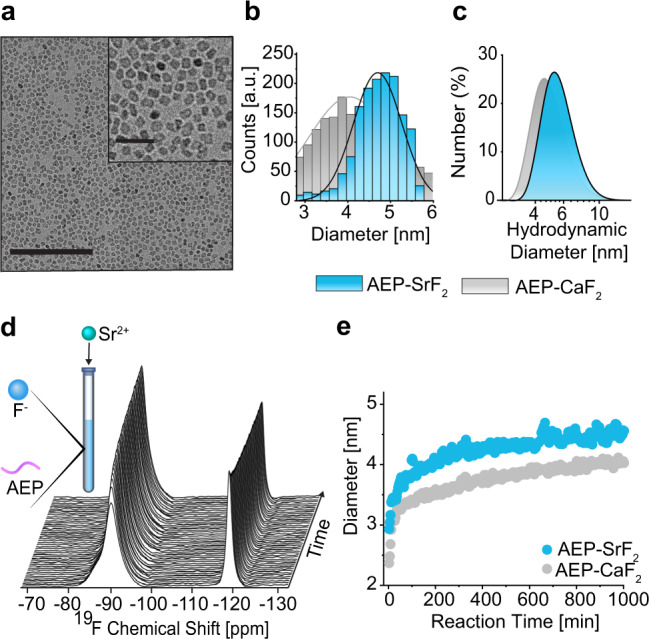
AEP-SrF_2_ NC formation. **a** Cryo-TEM of AEP-SrF_2_ in water (1000 min after reaction initiation). **b**, **c** Size distributions of AEP-SrF_2_ (light blue) and AEP-CaF_2_ NCs (gray) as obtained from (**b**) cryo-TEM and (**c**) DLS. **d** Stack plot of the real-time ^19^F-NMR spectra of AEP-SrF_2_ synthesis. **e** Size evolution of the diameter of NCs in water throughout the reaction process for AEP-SrF_2_ (light blue) and for AEP-CaF_2_ (gray), as derived from Eq. (1).

The generality of the proposed *in-situ* ^19^F-NMR for studying NC formation and growth was demonstrated also for SrF_2_ NCs (Fig. 3 and Supplementary Fig. 11). Compared to CaF_2_ NCs, the SrF_2_ NCs were larger, as determined by cryo-TEM (Fig. 3a, b) and DLS (Fig. 3c). The difference in size in the sub-nm range could be clearly depicted by the HR-^19^F-NMR approach (Fig. 3d, e), and the method was applied to monitor, in situ and in real time, the formation of AEP-SrF_2_ NCs. Notably, at three minutes after reaction initiation, the SrF_2_ NCs were much larger than the CaF_2_ NCs (2.9 nm vs. 2.3 nm, respectively), a size difference that was preserved throughout the reaction. Similar to the case of CaF_2_, the temporal evolution of SrF_2_ NCs is characterized by an initial fast growth followed by a moderate leveling-off of the size with time. Importantly, for both investigated nanofluorides, the concentration of the NCs in the solution decreased in parallel to their growth (Figs. 2d, e, 3e, and Supplementary Fig. 11b). To further elucidate this formation mechanism, we monitored the ligands during NC synthesis by way of in situ liquid-state ^31^P-NMR (Supplementary Fig. 12). The ^31^P-NMR peak of free AEP in solution is a clear narrow triplet. Upon the initial formation of NCs, this triplet broadens probably due to the restricted mobility of the bound ligands to the numerous very small NCs that had formed. As the reaction progresses, when the number of NCs in the solution drops (Fig. 2e and Supplementary Fig. 11b), free AEP-ligands are re-released to the solution and their ^31^P-NMR peak narrows again due to their faster tumbling rates.

### The role of ligands in the NC growth pathways

**Fig. 4:**
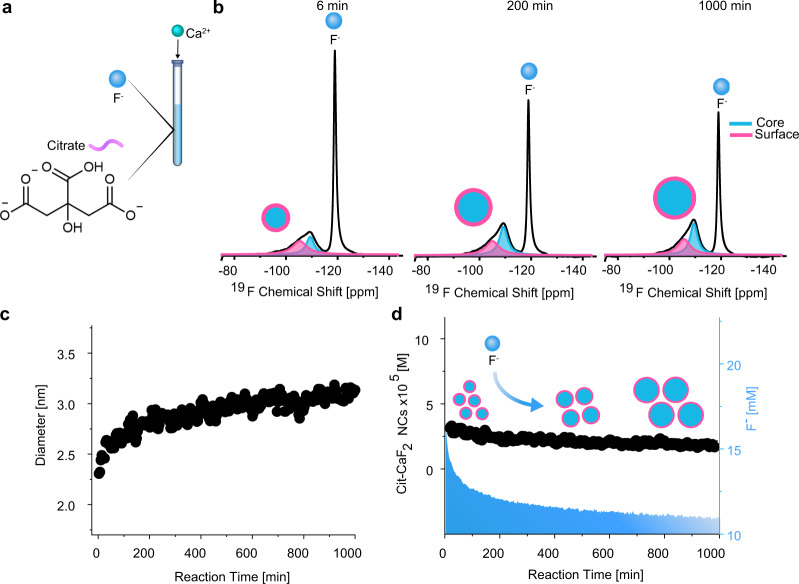
Ligand effect on CaF_2_ NC’s growth mechanism. **a** Schematic illustration of the real-time synthesis of Cit-CaF_2_ NCs in water and of the molecular structure of citrate. **b** ^19^F-NMR spectra from three different time points during the reaction (6 min, 200 min, and 1000 min), showing the deconvoluted signals of ^19^F atoms on the surface (pink) and in the core (blue) in addition to the peak at −120 ppm assigned to free F^-^ ions. **c** Size evolution of Cit-CaF_2_ NCs in water from a real-time, high-resolution ^19^F-NMR data set. **d** Concentration of Cit-CaF_2_ NCs (multiply by 105 in the graph) in water (left axis, black) or F^-^ ion consumption (right axis, light blue) as a function of the reaction time.

It is well known that ligands regulate NC formation mechanisms and dynamics, as they were found to be key elements in stabilizing both the reaction intermediates^8,49^ and formed colloids^50,51^. Specifically, differences in NC growth mechanisms have been attributed to the different affinities of the ligands to the cations^52,53^ and to their role in stabilizing the formed colloids^54^. Therefore, we decided to study the growth dynamics of CaF_2_ NCs in water using a different capping ligand. In contrast to AEP (Fig. 1a), negatively charged ligands may hinder NC interactions at the NC’s initial growth stage, due to electrostatic repulsion, which could lead to a different growth mechanism^55^. Interestingly, applying in situ ^19^F-NMR to the synthesis of CaF_2_ NCs in the presence of the negatively charged citric acid (citrate at pH = 7, Fig. 4a) as the capping ligand revealed a different growth mechanism than that observed for AEP-CaF_2_ (compare Fig. 2 and Fig. 4). We detected in the representative ^19^F-NMR spectra obtained at different reaction time points (i.e., 6 min, 200 min, and 1000 min, Fig. 4b) a notable consumption of free fluoride (peak at −120 ppm). This observation implies a growth mechanism that differs from particle-coalescence found for AEP-CaF_2_. Assigning the ^19^F-NMR peaks at −105 and −109 ppm to the fluorides of the surface and of the core of Cit-CaF_2_NCs, respectively, allows to determine their size evolution (using Eq. (1) and Supplementary Note 1), revealing their continuous growth (Fig. 4c), while their final size was further verified by TEM (Supplementary Fig. 13).

**Fig. 5:**
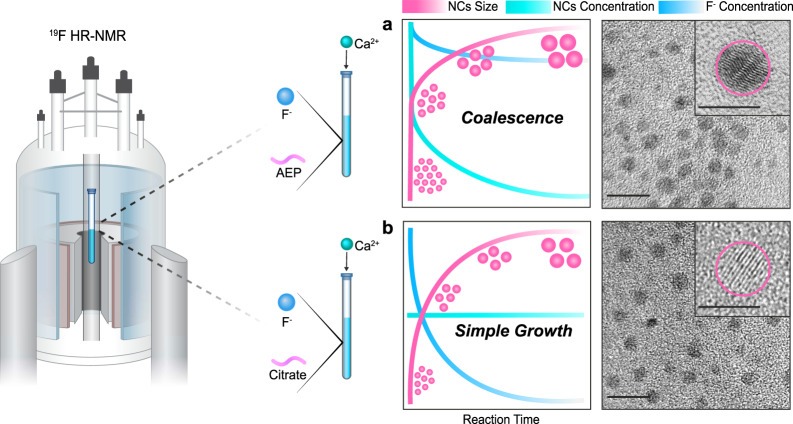
Ligand effect on CaF_2_ NC’s growth mechanisms and crystallographic properties. Schematic illustration of CaF_2_ NC formation with different ligands; (**a**) AEP and (**b**) Citrate. The NCs’ size (in pink), concentration (in turquoise) and F^-^ ion concentration in solution (in blue). HR-TEM images of AEP-CaF_2_ NCs presenting twinning defects (inset, in **a**) and of Cit-CaF_2_ NCs presenting a crystalline morphology (inset, in **b**).

Importantly, contrary to that observed for AEP-CaF_2_ NCs (Fig. 2e), the concentration of the growing Cit-CaF_2_ NCs was near-constant throughout the synthesis (Fig. 4d, black). This observation, in addition to the significant depletion of the F^-^ anion throughout Cit-CaF_2_’s growth (which is negligible for AEP-CaF_2_, Supplementary Fig. 5), suggests a different growth mechanism. While AEP-CaF_2_ NCs grow mostly by coalescence, as reflected by the pronounced reduction in their concentration (Fig. 2), Cit-CaF_2_ NCs primarily grow by the classical simple-growth mechanism^10^. This result emphasizes another advantage of our proposed approach. Besides its capability to determine NCs’ size evolution, it can also differentiate between two different growth mechanisms as schematically depicted in Fig. 5 (coalescence vs. simple classical growth, Fig. 5a, b). Nevertheless, it is important to mention here that although the proposed in situ NMR approach allowed to investigate NC growth pathways, it failed to pick-up early and rapid nucleation phases.

Finally, we studied the crystallographic features and spin-lattice relaxation properties of AEP-CaF_2_ and Cit-CaF_2_ NCs, as it was shown that the growth mechanism as well as the properties of the capping ligands impact the obtained architectures and characteristics of as-synthesized NCs^56,57^. Interestingly, HR TEM images of the coalesced AEP-CaF_2_ NCs showed crystallographic twinning defects for majority of the NCs (Fig. 5a and Supplementary Fig. 14), which were not observed in the crystalline Cit-CaF_2_ NCs (Fig. 5b and Supplementary Fig. 14). In addition, the longitudinal relaxation time T_1_ of the NCs’ fluoride content was shorter in AEP-CaF_2_ NCs as compared to that of Cit-CaF_2_ NCs (Supplementary Fig. 15). Such shorter T_1_ values of the defected NCs correlate with previous reports where mechanical stress-mediated lattice distortions of CaF_2_ powders resulted in significant reduced T_1_ values^58^, and should play a critical role in their future design as MRI tracers^59^. Our presented method for elucidating the contribution of the capping ligands to the nanofluoride’s characteristics could be used for tuning them as functional nanomaterials for a variety of uses, as both their crystallinity^60^ and relaxation times^59^ were found to be key features in their performances.

In summary, we show here an approach for the real-time monitoring of NC evolution in solution based on the ability to detect in situ NMR signals of the elements of as-synthesized nanoformulations. The demonstrated ability to accurately determine the average NC diameter at each timepoint during the synthesis has provided us with valuable insight into NC growth mechanisms in solution. We found that in the presence of a phosphate-based ligand, NCs grow predominantly via particle coalescence, while a carboxylate-based ligand leads to a simple-growth mechanism of the same material. Each pathway yields different crystallographic features, and, consequently, distinct relaxation properties, highlighting the link between crystal growth dynamics, structure, and function. These findings should have numerous implications in the growing field of nanofluorides^23–25,27,59,61^ but also for other types of nanomaterials^62–66^ where the crystal properties determine the NC functionality. The generality of our NMR-based approach for revealing in-depth mechanistic insights of fluoride-NCs, is highlighted by the similar pattern of the NC evolution profiles found for other types of NCs studied by other in situ methods^10,46,47^. Therefore, we envision that the fundamental principles in NCs growth underlined here have the potential to serve as a foundation for future studies of other types of materials. Finally, the accessibility of high-resolution NMR spectrometers, in addition to the versatility of NMR acquisition schemes and the ability to study a variety of nuclei (beyond ^1^H and ^19^F), could be implemented to elucidate the growth mechanism from a perspective different from those provided by existing in situ techniques.

## Supplementary information

Supplementary Information

## Data Availability

The data that support the findings of this study are available from the corresponding author upon reasonable request.
