# Supplementary material for: In situ NMR reveals real-time nanocrystal growth evolution via monomer-attachment or particle-coalescence
Source: Nat Commun. 2021 Jan 11;12:229. doi: 10.1038/s41467-020-20512-6 (PMC7801738; doi:10.1038/s41467-020-20512-6)
Supplement: Supplementary file 1 — Supplementary Information [file 41467_2020_20512_MOESM1_ESM.pdf]

Supplementary Information

In-situ NMR reveals real-time nanocrystal growth evolution via monomer-attachment or particle-coalescence

Reut Mashiach[1], Haim Weissman[1], Liat Avram[2], Lothar Houben[2],
Olga Brontvein[2], Anna Lavie[3], Vaishali Arunachalam[3], Michal Leskes[3],
Boris Rybtchinski[1], Amnon Bar-Shir[1*]
[1]Department of Organic Chemistry, [2]Department of Chemical Research Support,
[3]Department of Materials and Interfaces, Weizmann Institute of Science,
Rehovot, 7610001, Israel

*Corresponding Author: amnon.barshir@weizmann.ac.il

Methods

Synthesis of AEF_2 NCs coated with AEP

A 25 ml solution of AEP (0.17M) and NaF (0.08M) in ddH_2O (milli-Q grade) pH=7, (neutralized with ammonium hydroxide) was heated to 60°C under stirring. Then, 1 ml $M(NO_3)_2$ ($M=Ca^{+2}/Sr^{+2}$) (0.04M) was rapidly added to the hot solution. After 100 min at 60°C, the solution was removed from the heating plate and cooled to room temperature. Then, the solution was centrifuged and washed with ethanol/ddH_2O.

For *in-situ* synthesis, a 1 ml solution of AEP/citrtic acid (0.17M) and NaF (0.08M) in ddH_2O pH=7 (neutralized with ammonium hydroxide) was placed inside a 5 mm NMR tube. Then, $M(NO_3)_2$ (0.04M) was injected and the tube was inserted into the NMR spectrometer at 25°C.

In-situ NMR

In-situ NMR experiments were performed with the sample temperature stabilized at 25°C. ^{19}F-NMR (376.7 MHz) spectra were acquired with a 30° flip angle, according to Ernst theory. Ernst angle conditions were used in order to maximize SNR in a given time unit and to shorten the experiment time and, thus, to allow optimal temporal resolution . The conditions were determined according to the equation $\cos(\alpha)=e^{-TR/T_1}$ with TR and according to the T_1 relaxation properties of the studied AEP-MF_2 (M = Ca^{2+} or Sr^{2+}) and Cit-CaF_2 NCs. First, T_1=2.7sec was found for AEP-MF_2 (M = Ca^{2+} or Sr^{2+}) and T_1=4sec for Cit-CaF_2 from the inversion recovery (IR) experiment. For the in-situ experiment of AEP-MF_2 (M = Ca^{2+} or Sr^{2+}) the experiment time was set to 3 min and resulted in the following α and TR values: $\alpha=30^o$ and TR=0.39 sec. For Cit-CaF_2 experiment time was set to 3:40 min and resulted in $\alpha=30^o$ and TR=0.57 sec. Sequential ^{19}F-NMR spectra were acquired with 240 scans for all experiments. At the end of all real-time syntheses (1200 min), we obtained an additional ^{19}F spectrum of the end product together with a capillary filled with triflouroactetae (with a known F- concentration) for an appropriate qualitative and quantitative calibration between the different experiments.

^{31}P *in-situ* NMR were acquired according to the following parameters: T_1=3 sec for AEP-CaF_2/SrF_2 ligands, TR=15 sec, NS=12.

Solid State NMR

^{19}F solid state NMR experiments were performed on a 9.4 T (400 MHz) AvanceIII spectrometer using a Bruker 1.3 mm double-resonance probe at magic angle spinning (MAS) rate of 60 kHz. Spectra were recorded within 2 scans using a single pulse excitation of 1.9μs (133 kHz nutation frequency) with a relaxation delay of 30 s between each scan. The relaxation delay was longer than 5 times the longitudinal relaxation time measured by saturation recovery. The chemical shift was references with respect to LiF standard set at -204 ppm.

Line Fitting Procedure

The high-resolution ^{19}F-NMR spectrum of AEP-CaF_2 NCs shown in Fig 2b was decomposed into three Lorentzian peaks centered at -105, -109 and -120 ppm using non-linear least squares fit from MATLAB's curve fitting toolbox. The goodness of fit was evaluated using R-square which ranged from 0.96 to 0.98. The integrated area under each Lorentzian peak was obtained using MATLAB's inbuilt function. The ^{19}F-NMR spectra of AEP-SrF_2 NCs (Fig 3d) and Cit-CaF_2 NCs (Fig 4b) were similarly decomposed and their respective integrated area evaluated.

X-Ray Diffraction (XRD)

Diffraction measurements were carried out with reflection geometry using an Ultima III (Rigaku, Japan) diffractometer equipped with a sealed, Cu anode X-ray tube operating at 40 kV and 40 mA. A bent graphite monochromator and a scintillation detector were aligned in the diffracted beam. $\theta/2\theta$ scans were performed under specular conditions in the Bragg-Brentano mode with variable slits. The 2θ scanning range was 10–120 degrees, with a step size of 0.025 degrees and a scan speed of 0.5 degrees per minute. Phase analysis was performed using the PDF-4+ 2015 database (ICDD) and Jade 9.5 software (Materials Data, Inc.).

Dynamic Light Scattering (DLS)

The diameter and size distribution of the obtained nanoparticles were evaluated by dynamic light scattering, Malvern Nano-ZS. Measurements were taken in a 12.5 mm diameter plastic cuvette according to the appropriate solvent viscosity (for water= 0.8827 cp).

Transmission Electron Microscopy (TEM)

STEM and hyperspectral EDS data (Fig.1) were taken in a probe and image-corrected Themis-Z transmission electron microscope (Thermo Fisher Scientific Microscopy Solutions, Hillsboro, USA) equipped with a Super-X SSD detector at an acceleration voltage of 200 kV. Dry samples were prepared by drop cast deposition on an ultra-thin carbon support foil. TEM and high-resolution TEM (HRTEM) images (Fig.5) were acquired with a JEOL JEM 2100 high-resolution electron microscope, Gatan Digital Micrograph. Accelerating voltage was 200 kV, a beam source of an LaB6 thermal emission, and a bottom-mount CCD camera (Gatan Ultra scanXP 2k x 2k).

Cryo-TEM

For the preparation of the cryo-TEM specimens, a 6.5 µL sample (according to the appropriate synthesis upon injection of the second precursor) was applied to a 200-mesh copper grid coated with holey carbon (Pacific Grid-Tech supplies). The grid was subjected to a 1 min glow discharge before sample application. Samples were blotted at 22°C and 95% relative humidity and then plunged into liquid ethane using a Leica EM-GP Automatic Grid Plunger. Specimens were equilibrated at -178°C in the microscope prior to imaging. Cryo-TEM was performed on a Tecnai F20 twin-transmission electron microscope (Thermo-Fisher corporation) operating at an acceleration voltage of 200 kV. Cryogenically preserved samples were loaded into a Gatan 626 cryoholder. Low-dose TEM images were recorded with a Gatan US4000 CCD. Two different samples were prepared: 1. AEP-CaF_2, 6 min after the injection of Ca^{+2} into a solution containing both AEP and F^- (according to the synthesis described above). 2. AEP-SrF_2, 1000 min after the injection of Ca^{+2} into a solution containing both AEP and F^- (according to the synthesis described above). Cryo-TEM was performed on a Talos Arctica (TFS) TEM at an acceleration voltage of 200 kV. Low-dose cryo-TEM images were recorded with a Gatan OneView 4kx4k CMOS camera. Two different samples were prepared at different stages of AEP-CaF_2 formation: 200 min and 1000min after the injection of Ca^{+2} into a solution containing both AEP and F^- (according to the synthesis described above).

Supporting Figures

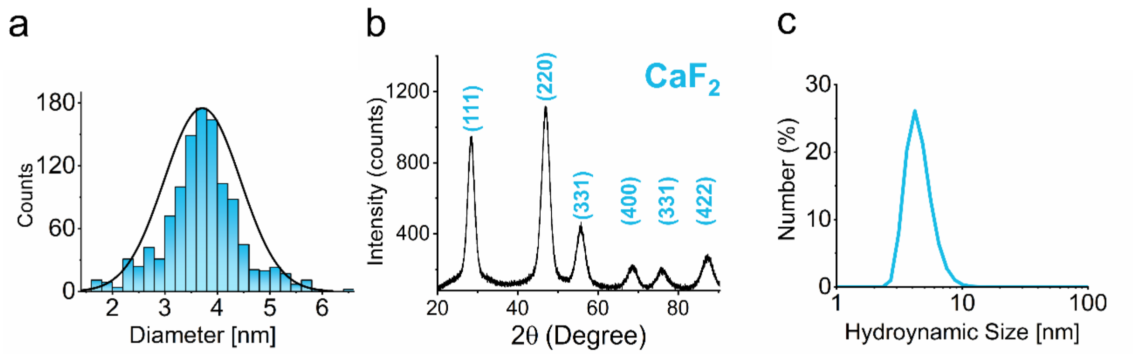

Supplementary Figure 1. Characterization of the CaF_2-AEP NC (a) particle size distribution histogram by TEM (D_{TEM}=3.7±0.6 nm) (b) XRD patterns of the crystalline CaF_2. (c) Hydrodynamic size distribution with DLS (D_{DLS}=4.6 ± 1. 2nm).

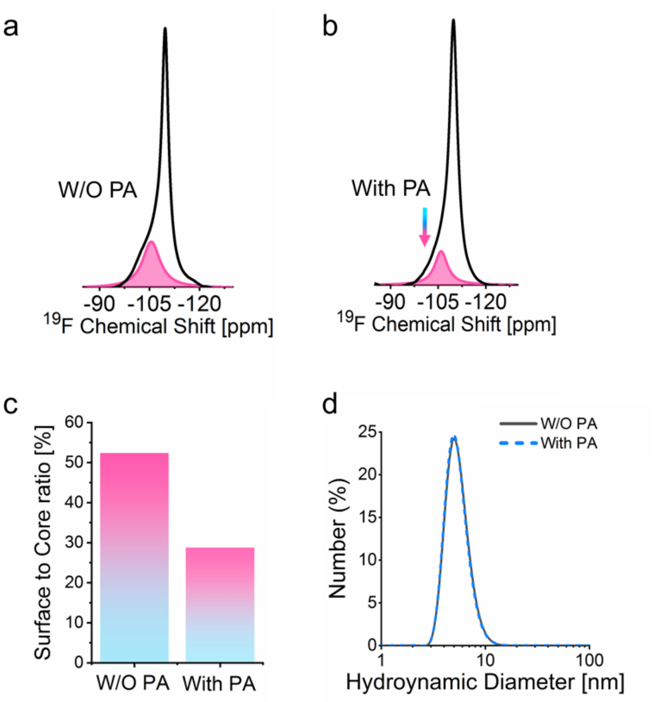

Supplementary Figure 2. The effect of paramagnetic agent (PA) on the ^{19}F-NMR spectra of AEP-CaF_2 NCs in water. ^{19}F -NMR spectra of AEP-CaF_2 NCs in water and the convolved peak area at -105 ppm, assigned to the surface atoms before (a) and after (b) the addition of $SmCl_3$ salt as the PA to the studied solution. (c) The ratio of the integrated area of the ^{19}F-NMR peaks assigned to surface (-105 ppm) and core (-109 ppm) atoms, with and without the PA. (d) The hydrodynamic diameter (by DLS) of AEP-CaF_2 NCs in water with and without the addition of $SmCl_3$ salt as the PA showing no change in the NCs' size.

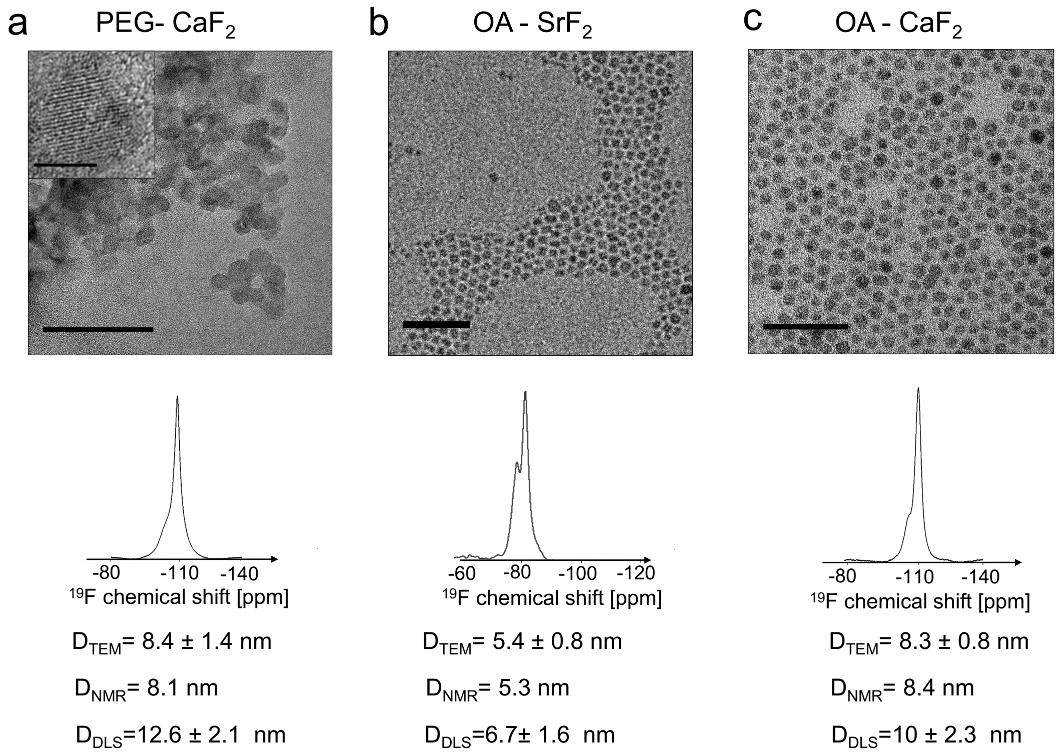

Supplementary Figure 3. MF_2 ($M=Ca^{2+}$ or Sr^{2+}) NCs and their size measurements with TEM ^{19}F NMR and DLS. (a) PEG-CaF_2 in water (scale bar 50nm, inset is 5nm). (b) OA-SrF_2 in cyclohexane (scale bar 50nm). (c) OA-CaF_2 in cyclohexane (scale bar 100nm).

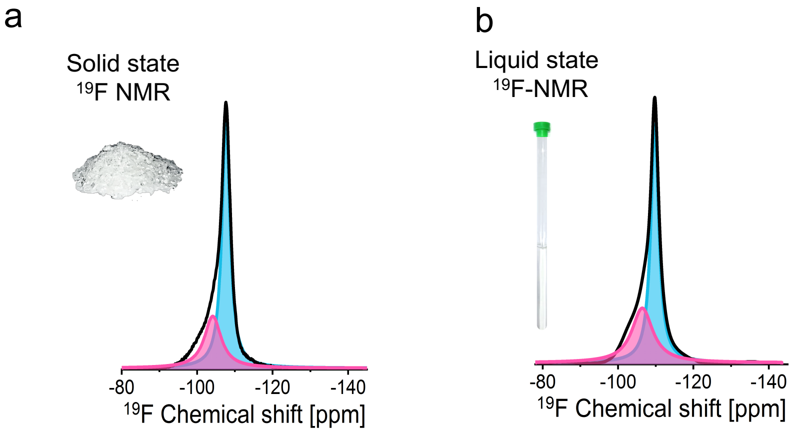

Supplementary Figure 4. ^{19}F NMR of AEP-CaF_2 NCs. (a) Solid state NMR of a dried powder with MAS of 60 kHz. (b) High resolution ^{19}F NMR of NCs in water.

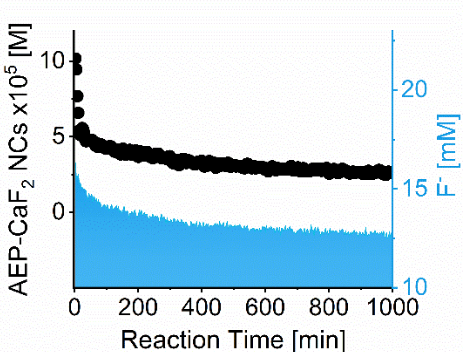

Supplementary Figure 5. Real-time ^{19}F NMR of F^- concentration during the formation of AEP-CaF_2. Concentration of AEP-CaF_2 NCs in water (left y-axis, black) or F^- ion consumption (right axis, light blue) as a function of the reaction time.

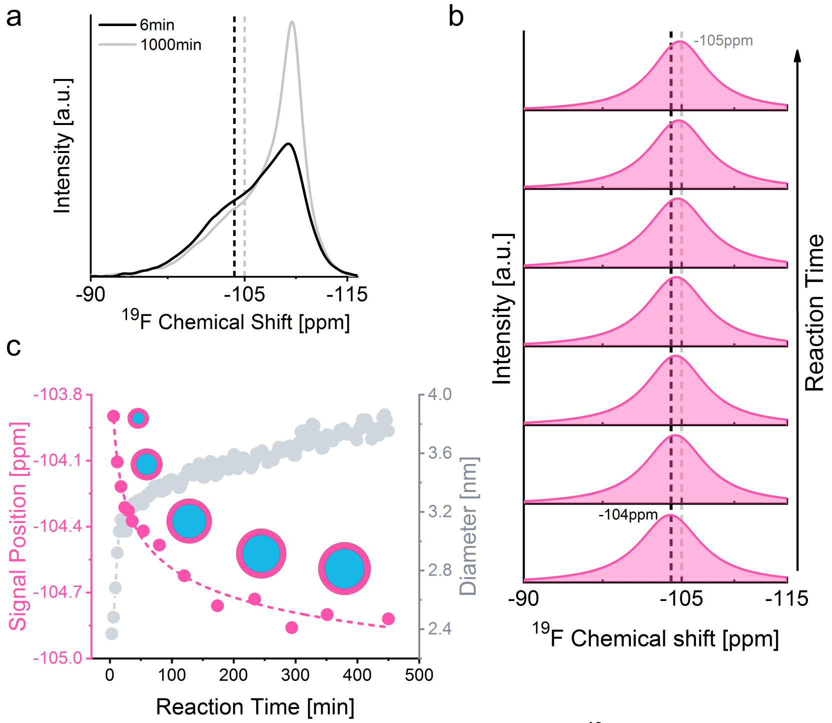

Supplementary Figure 6. Temporal evolution of the ^{19}F-NMR spectra as a function of the AEP-CaF_2 reaction time. (a) Representative ^{19}F-NMR spectra of the reaction mixture of AEP-CaF_2 6 min (black) and 1000 min (grey) after reaction initiation. (b) The temporal evolution of the deconvolved peak assigned to the surface fluorides (at -105 ppm) throughout the formation of AEP-CaF_2 NCs (from bottom to top: 6, 36, 54, 81, 120, 234 and 444 min after reaction initiation). Dashed lines in (a) and (b) are reference lines representing the chemical shift values at -104 ppm (black) and -105 ppm (grey). (c) Left Y-axis: The chemical shift of the deconvolved peaks assigned to the surface fluorides as a function of the reaction time. Right Y-axis: The calculated diameter of the newly formed AEP-CaF_2 NCs as a function of the reaction time.

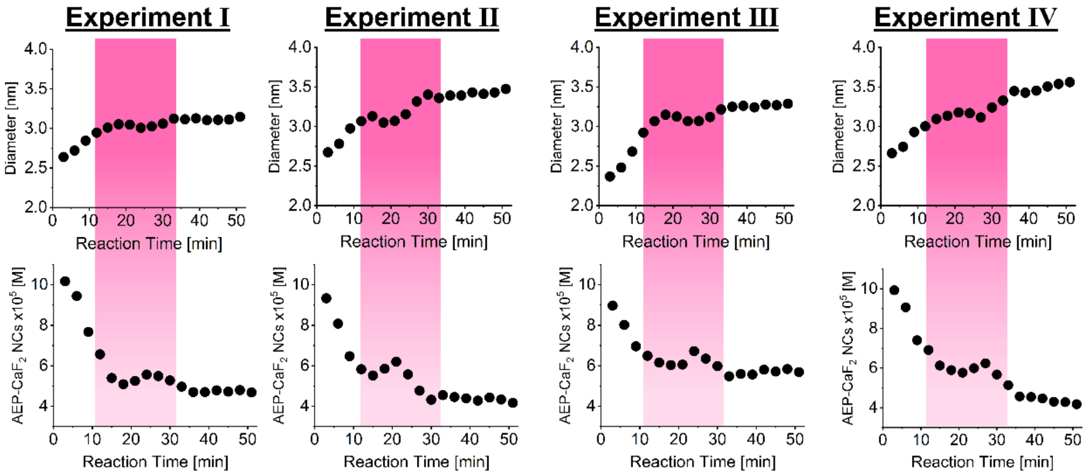

Supplementary Figure 7. Real-time, high-resolution ^{19}F-NMR of AEP-CaF$_2$ NC evolution in water (first 60 min of the reaction). Results from four independent experiments showing the evolution of the AEP-CaF$_2$ NC diameter (top) and their concentrations (bottom) in water throughout the reaction process.

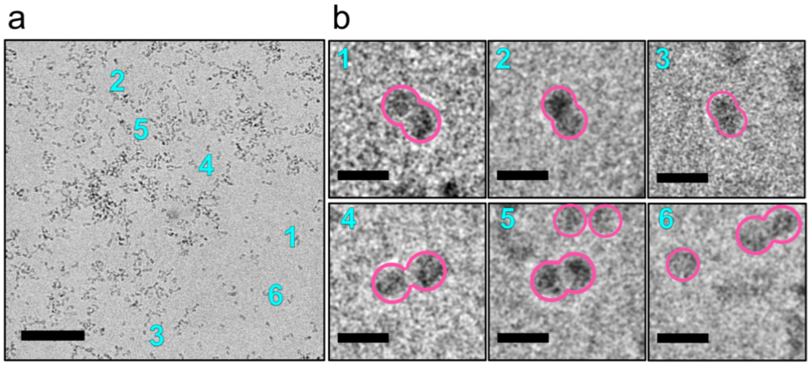

Supplementary Figure 8. Cryo-TEM of AEP-CaF$_2$ NCs in water (25 min after reaction initiation); (a) Cryo-TEM image with six (1-6) notations of regions with coalescing NCs, scale bar=50 nm**.** (b) Zoom-in of regions 1-6 shown in (a), scale bar=5 nm.

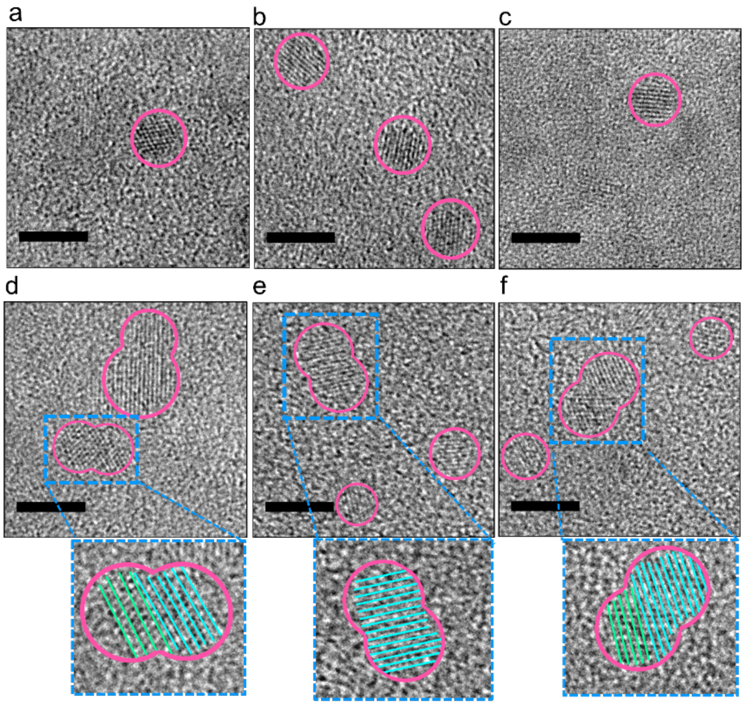

Supplementary Figure 9. HR-TEM image of AEP-CaF_2 NCs (25 min after reaction initiation). (a)-(c) Single crystalline particles detected with d spacing of d = 0.315 nm of CaF_2 NCs. (d)-(f) Detected coalesced NCs demonstrating the fusion of the crystalline cores. The inset at the bottom of the lines in green and turquoise illustrates the CaF_2 lattice planes of the two NCs coalesced to form boundary defects.

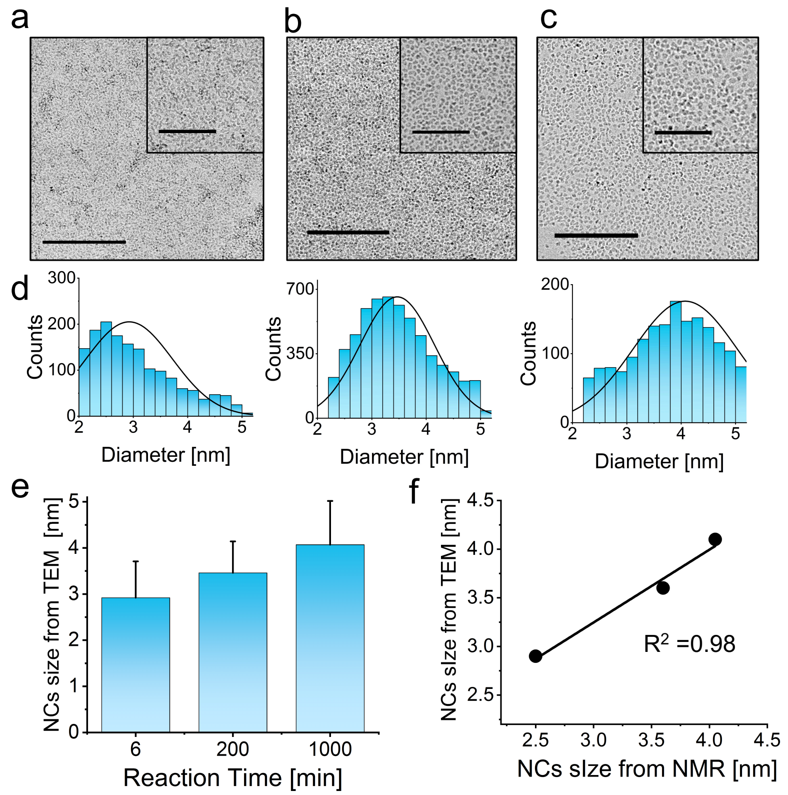

Supplementary Figure 10. Cryo-TEM of AEP-CaF_2 formation. Cryo-TEM of samples taken from the reaction mixture after (a) 6 min, (b) 200 min and (c) 1000 min. Scale bar 100 nm (inset 50 nm). (d) Particle size distribution histogram of the of AEP-CaF_2 NC by Cryo-TEM at different time point of the synthesis; (e) AEP-CaF_2 NC diameters, as calculated from the Cryo-TEM images (a-c),(a) 6min, D= 2.9 ±0.8nm, (b) 200min, D= 3.4 ±0.7nm and (c)1000min, D= 4.1 ±0.9nm. (f) Correlation between the NC size as measured from Cryo-TEM images and from ^{19}F-NMR spectra.

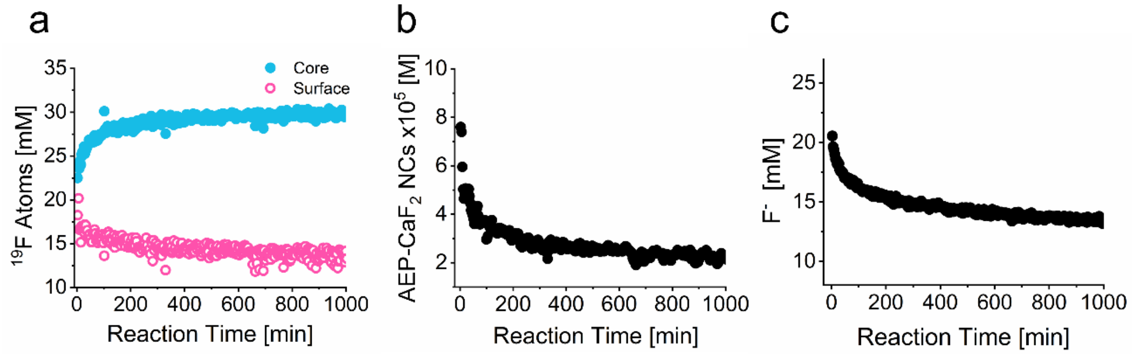

Supplementary Figure 11. Real-Time ^{19}F HRNMR synthesis of AEP-SrF_2 NCs in water (a) ^{19}F core atoms calculated from the deconvoluted peak area at -89ppm, every 3 minutes (in light blue) and ^{19}F surface atoms calculated from the convoluted peak area at -87ppm, every 3 minutes (in pink) (b) The concentration of AEP-SrF_2 NCs in solution with time calculated from Supplementary Note 2 (c) ^{19}F NMR monitoring the concentration of F^- (peak at -120ppm) in solution during the synthesis of AEP-SrF_2 NCs.

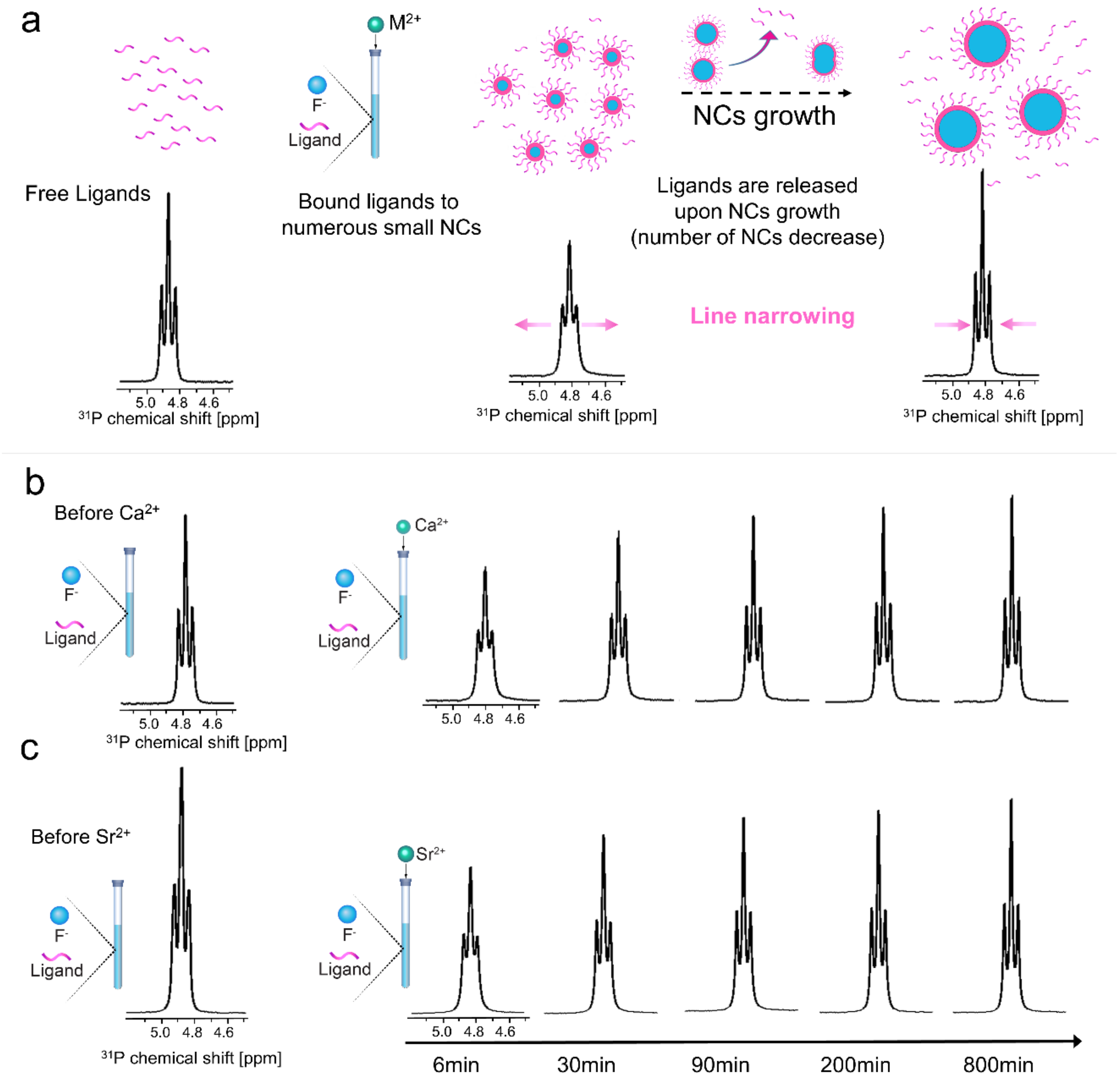

Supplementary Figure 12. ^{31}P-NMR monitoring the AEP ligand in real time. (a) Schematic illustration of the ligands release during the NCs growth and the appearance of the AEP triplet before, during and at the end of the synthesis. (b) ^{31}P-NMR of the AEP ligand before the addition of Ca^{2+} and after the AEP-CaF_2 NCs reaction initiation. (c) ^{31}P-NMR of the AEP ligand before the addition of Sr^{2+} and after the AEP-SrF_2 NCs reaction initiation.

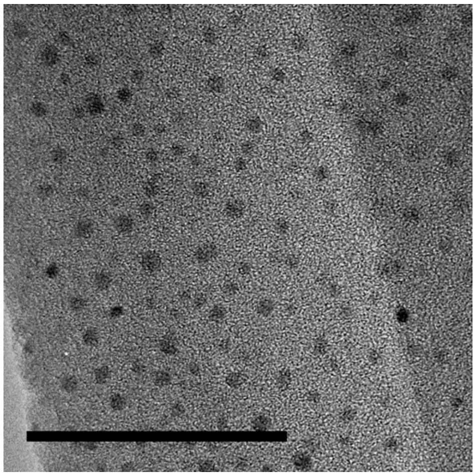

Supplementary Figure 13. TEM image of Cit-CaF_2 after 1000min of reaction with a particle size distribution from TEM of D= 3.2±1.0 nm.(Scale bar 50nm).

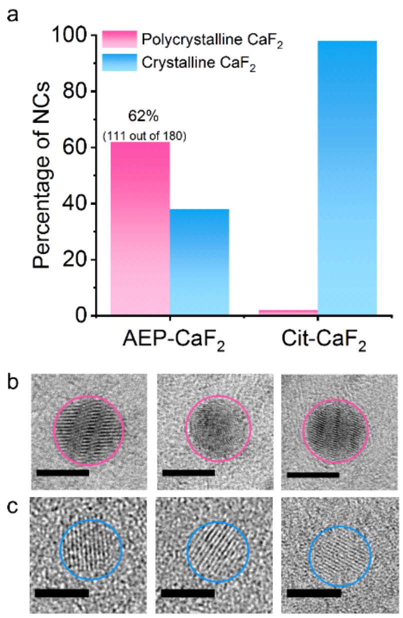

Supplementary Figure 14.Statistical analysis of CaF_2 crystallinity (a) Statistical analysis of defected vs. crystalline NCs detected with HR-TEM of AEP-CaF_2 and Cit-CaF_2 NCs. HR-TEM images of single particles either (b) AEP-CaF_2 NCs or (c) Cit-CaF_2 NCs.

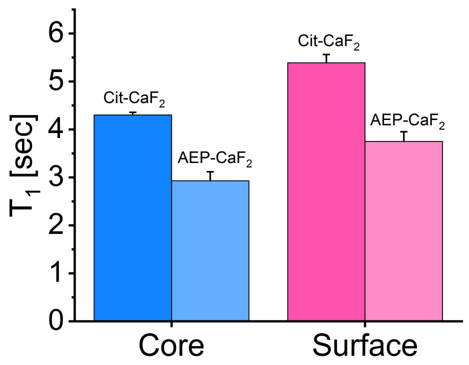

Supplementary Figure 15. ^{19}F T_1 relaxation properties of CaF_2 NCs in water. Cit-CaF_2 T_1 relaxation properties of the deconvolved NC components (core in blue and surface in pink). AEP-CaF_2 T_1 relaxation properties of the deconvolved NC components (core in light blue and surface light ligth pink).components (core in light blue and surface in light pink).

Supplementary Notes

Supplementary Note 1; Derivation of Equation 1.

Calculating the size of the NCs from the ratio between the convoluted NMR peaks of the surface ^{19}F atoms and core ^{19}F atoms.

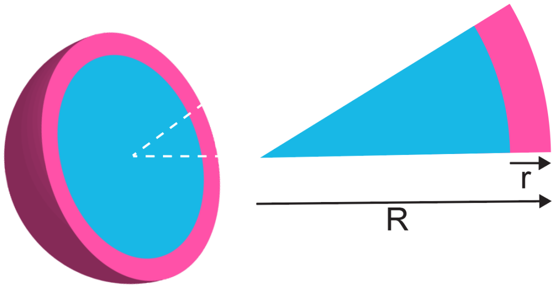

$$\frac{^{19}F_{surface\ atoms}}{^{19}F_{core\ atoms}} = \frac{V_{surface} \times d}{V_{core} \times d} = \frac{V_{NC} \times d - V_{Core} \times d}{V_{core} \times d} = \frac{\frac{4}{3}\pi R^3 - \frac{4}{3}\pi (R-r)^3}{\frac{4}{3}\pi (R-r)^3}$$

$$= \frac{R^3}{(R-r)^3} - 1$$

$^{19}F_{surface\ atoms}$ *are calculated from the deconvulated area 1D NMR spectrum at* $-105\ ppm$

$^{19}F_{core\ atoms}$ *are calculated from the deconvulated area 1D NMR spectrum at* $-109\ ppm$

d – *density of* MF_2 *NCs*

$V_{surface}$ – *volume of outer sphere (pink)*

V_{core} – *volume of inner sphere (ligth blue)*

V_{NC} – *volume of NCs*

r – *length of the outer surface, attributed to the ionic diameter of fluorine atoms* ($r = 0.266\ nm$ [1])

Supplementary Note 2; Derivation of Equation 2.

Calculating the concentration of NCs in solution at each reaction time point based on real-time ^{19}F NMR spectra.

The total number of NCs in solution;

$$N(t) = \frac{Total\ number\ of\ ^{19}F\ atoms\ in\ NCs(t)}{Number\ of\ ^{19}F\ atoms\ per\ NC(R(t))} = \frac{(\mathbf{1})}{(\mathbf{2})}$$

$(\mathbf{1})$

The total number of ^{19}F atoms in a NC is the sum of the deconvoluted peaks assigned to the core and surface atoms at each time point, based on the 1D ^{19}F NMR spectra.

$$(\mathbf{1}) = {}^{19}F_{core}\ atoms\ (t) + {}^{19}F_{surface}\ atoms\ (t)$$

$${}^{19}F_{core/surface}\ atoms\ (t) = C_{core/surface}(t) \times V_{reaction} \times N_A$$

$C(t) - concentration\ of\ ^{19}F\ atoms\ (core\ or\ surface)\ measured\ from\ the\ calibrated$

$and\ convoluted\ integration\ area\ at\ each\ time\ point.$

$V_{reaction} - reaction\ volume\ of\ 1\ ml.$

$N_A - Avogadro's\ constant\ (6.023 \times 10^{23})$

$(\mathbf{2})$

The number of ^{19}F atoms in NCs is a function of their radii at each time point and is also determined by the properties of MF_2 (CaF_2 or SrF_2) compounds, such as molecular weight ($M_{w_{MF_2}}$) or atom densities(d_{MF_2}).

$$moles\ of\ atoms\ per\ MF_2\ NC\colon\ nMF_2 = \frac{d_{MF_2} \times V_{MF_2}}{M_{w_{MF_2}}}$$

$$d_{CaF_2} = 3.18\ {}^{gr}/_{cm^3} \quad ,\ M_{w_{CaF_2}} = 78.07\ {}^{gr}/_{mol}$$

$$d_{SrF_2} = 4.24\ {}^{gr}/_{cm^3} \quad ,\ M_{w_{SrF_2}} = 125.62\ {}^{gr}/_{mol}$$

$$Volume\ of\ NCs\colon\ V_{MF_2} = \frac{4\pi\ (R(t))^3}{3}$$

$Since\ each\ MF_2\ has\ 2\ ^{19}F\ atoms\ per\ NC, the\ number\ of\ ^{19}F\ atoms\ per\ MF_2\ NC\ can\ be\ calculated\ from:$

$$2 \times nMF_2 \times N_A$$

$$Number\ of\ ^{19}F\ atoms\ per\ CaF_2 = 204.7 \times R(t)^3$$

$$Number\ of\ ^{19}F\ atoms\ per\ SrF_2 = 170.3 \times R(t)^3$$

Calculating the concentration of NCs(t), C_{NC}, in solution [M].

$$C_{NC} = \frac{N(t)}{N_A \times V_{reaction}} \qquad (Supplementary\ \text{Equation 2})$$

$N(t) - number\ of\ NCs\ in\ solution$

$N_A - Avogadro's\ constant\ (6.023 \times 10^{23})$

$V_{reaction} - reaction\ volume\ of\ 1ml.$

Supplementary References

1. Haynes, W.M. *CRC handbook of chemistry and physics*, (CRC press, 2014).
